# Prescription Medication Use During Pregnancy and Postpartum in Sweden 2007 to 2022

**DOI:** 10.1002/pds.70473

**Published:** 2026-09-09

**Authors:** Essi Whaites Heinonen, Awad I. Smew, Silvia Segovia Chacón, Ulrika Nörby, Olof Stephansson, Anne K. Örtqvist, Cathrine Axfors, Pär Karlsson, Carolyn E. Cesta

**Affiliations:** ^1^ Centre for Pharmacoepidemiology, Division of Clinical Epidemiology, Department of Medicine Solna Karolinska Institutet Stockholm Sweden; ^2^ Department of Neonatology Karolinska University Hospital Stockholm Sweden; ^3^ Department of Perioperative Medicine and Intensive Care Karolinska University Hospital Stockholm Sweden; ^4^ Health and Medical Care Administration Region Stockholm Stockholm Sweden; ^5^ Division of Clinical Epidemiology, Department of Medicine Solna Karolinska Institutet Stockholm Sweden; ^6^ Department of Women's Health, Division of Obstetrics Karolinska University Hospital Stockholm Sweden; ^7^ Department of Obstetrics and Gynecology Visby County Hospital Visby Sweden; ^8^ Department for Women's and Children's Health Uppsala University Uppsala Sweden; ^9^ Meta‐Research Innovation Center at Stanford (METRICS), Stanford University School of Medicine Stanford California USA

**Keywords:** drug utilization, maternal age, medication, pharmacoepidemiology, postpartum, pregnancy

## Abstract

**Purpose:**

Medication use during pregnancy has increased worldwide. The aim was to investigate patterns of prescription medication use during pregnancy and postpartum in Sweden 2007–2022.

**Methods:**

We conducted a population‐based drug utilization study with linked data from the Swedish Medical Birth and Prescribed Drug Registers. From 90 days before pregnancy to 90 days after delivery, all dispensed prescriptions for each full‐level ATC code were identified. The proportion of women, by year and age, with ≥ 1 dispensed medication was calculated, as well as the number of different medications and dispensed prescriptions of structural teratogens and specific medication groups.

**Results:**

Overall, 1 065 593 (60.5%) women were dispensed ≥ 1 medication during pregnancy and 900 536 (51.1%) within 3 months postpartum. The proportion of women with ≥ 1 dispensed prescription in the postpartum period was stable but increased during pregnancy from 57.1% in 2007 to 67.5% in 2022, with the largest increase in women ≥ 40 years. The proportion using ≥ 5 different medications increased, while the proportion with a recognized or suspected structural teratogen in the first trimester remained low (< 1%). There was a notable decrease in the use of antibiotics and prescribed analgesics, and an increase in antiemetics, psychotropics, acetylsalicylic acid, anticoagulants, antihypertensives, and antidiabetics.

**Conclusion:**

Medication use during pregnancy in Sweden increased from 2007 to 2022, particularly among women ≥ 40 years, while postpartum use remained stable. Shifts in the use of specific medication groups highlight evolving maternal characteristics and clinical practice, as well as the importance of continuous surveillance and investigation of medication safety.

## Key Points

- In Sweden 2007–2022, the proportion of women who were dispensed at least one prescription medication during pregnancy increased from 57% to 68%, with the largest increase among women ≥ 40 years, while postpartum use remained stable at 51%–54%.
- The proportion who were dispensed recognized and suspected structural teratogenic medications in the first trimester remained below 1%.
- We observed a decrease in the use of antibiotics and analgesics, and an increase in antiemetics, psychotropics, acetylsalicylic acid, anticoagulants, antihypertensives, and antidiabetics.
- Results highlight evolving maternal characteristics and clinical practices, and the need for surveillance and research to ensure medication safety for mothers and infants.

## Plain Language Summary

Over the past 15 years, the use of medications during pregnancy has increased in Sweden. In this study, we included all pregnancies from 2007 to 2022 and examined which medications women had dispensed during pregnancy and in the first 3 months after giving birth when breastfeeding decisions are made. We found that most pregnant women were dispensed at least one prescription; the proportion increased from 57% in 2007 to 68% in 2022, with the largest increases in the youngest and oldest women. The number of women taking multiple medications also rose. The most used medications during pregnancy were: antibiotics, medicines for nausea, and painkillers, and after birth: antibiotics, pain relievers, and blood‐thinners. The use of some medicines, such as those for nausea, mental health conditions, diabetes, blood‐thinning, high blood pressure, and diabetes, increased over time, whereas the use of antibiotics and painkillers after birth declined. Importantly, the proportion of women using medications with recognized or suspected risk of fetal malformations was very low. The trends in medication use reflect changes in maternal characteristics and evolving clinical needs for pregnant women. Findings highlight the importance of medication use surveillance and safety investigations for mothers and their infants.

## 1 Introduction

Most pregnant women use prescription medications and studies from both Europe and North America have demonstrated increases over time [1, 2, 3, 4, 5]. In Sweden, the last national overview of medication use during pregnancy was reported in 2011 [6]. Since then, both maternal characteristics and healthcare practices have evolved. There is therefore a need to update our understanding of medication use during pregnancy and postpartum.

Notably, women are having children later in their reproductive years, the prevalence of pre‐existing metabolic conditions (e.g., diabetes and obesity) has risen, and the use of assisted reproductive techniques is more common [7, 8, 9, 10]. New guidelines for prevention, detection, and management of high‐risk pregnancies, and pregnancy‐related conditions such as gestational diabetes and preeclampsia have been developed, including recommended use of acetylsalicylic acid [11, 12, 13]. Furthermore, observational studies in the last decade have provided robust evidence for short‐ and long‐term infant outcomes for some medications with previous safety concerns, such as antiemetics, psychotropics and antiseizure medications [14, 15, 16, 17, 18, 19, 20, 21].

Additionally, medication use during pregnancy often continues postpartum, and 93% of new mothers in Sweden initiate breastfeeding [22]. An updated understanding of medication use postpartum is therefore essential for identifying the most critical knowledge gaps in the safety evidence on medication use during breastfeeding [23, 24, 25, 26, 27, 28, 29, 30].

The objective of this study was to investigate time trends in prescription medication use during pregnancy and postpartum in Sweden from 2007 to 2022. Specifically, we aimed to describe, by year and age, the proportion of women using prescribed medications, the number of medications used, as well as the most commonly dispensed medications, first‐trimester use of recognized or suspected structural teratogenic medications, and patterns of use of specific medication groups of clinical interest.

## 2 Methods

### 2.1 Data Sources

We conducted a population‐based drug utilization study using Swedish national registers which contain prospectively collected health and demographic information on all residents. Via the unique personal identity number assigned to each resident at birth or immigration, individual‐level data was linked between the Medical Birth Register and the Prescribed Drug Register [31, 32, 33]. Initiated in 1973, the Medical Birth Register contains data from antenatal, delivery, and neonatal care for all live‐ or stillborn deliveries after 22 weeks gestation (> 99%) in Sweden (28 weeks gestation for stillbirths before 1 July 2008) [31].

Starting in July 2005, the Prescribed Drug Register contains data on all dispensed prescriptions in Swedish pharmacies, including full‐level (i.e., chemical substance) Anatomical Therapeutic Chemical Classification System (ATC) code [33]. ATC 2024 version was used and codes from chapters A through S were identified. Chapter V was not included since it contains non‐pharmaceutical products. The Prescribed Drug Register does not contain data on hospital‐administered medications or over‐the‐counter products. However, it does include data on dispensed prescriptions of vitamins and supplements which were included in this study since, when prescribed, they reflect a higher clinical need and are often used at higher doses.

### 2.2 Safety Classifications

Information on the safety classification according to the Swedish internet‐based clinical decision support systems for information on medication safety during pregnancy, *Janusmed fosterpåverkan* (“Drugs and Fetal Effects”), and during breastfeeding, *Janusmed Amning* (“Breastfeeding”), was identified (October 13–14, 2025) [34, 35]. These systems are maintained by Region Stockholm, are freely available on Janusmed.se, and are integrated in electronic health record systems for prescribers and in an electronic expert support tool for pharmacies. Both systems classify medications into three categories based on regular assessments of both the quality and quantity of available safety data for the individual substances (Table S1). Both *Janusmed fosterpåverkan* and *Janusmed amning* provide contextual information in narrative format with important considerations for a more complete risk evaluation of each medication.

### 2.3 Recognized and Suspected Structural Teratogenic Medications

A list of recognized or suspected structural teratogenic medications was derived from previous studies within the Nordic Pregnancy Drug Safety Study (NorPreSS) Consortium, re‐evaluated by the study team based on information provided in *Janusmed fosterpåverkan* and other latest available evidence up to September 2025, and harmonized with the latest list from the Innovative Medicines Initiative “ConcePTION” working group [34, 36, 37, 38, 39].

### 2.4 Study Population

All pregnant women with delivery dates between January 1, 2007, and December 31, 2022, were identified. Pregnancies with invalid or missing gestational length were excluded (*n* = 315). All eligible pregnancies for each woman were included in the study cohort. The unit of analysis was pregnancies, and to facilitate communication, “women”, “pregnant women”, and “pregnancies” are used interchangeably when describing the study population.

The date of the first day of the last menstrual period (LMP) was calculated by subtracting the gestational length in days from the delivery date. Gestational length in the Medical Birth Register is estimated using a hierarchy of: (1) ultrasound‐estimated gestational age, (2) maternal reported LMP, and (3) clinical examination at birth. Since the late 1990s, ultrasound‐dating is the basis for 85%–95% of pregnancies [31].

Pregnancy periods were: *pregnancy*: LMP to delivery‐1 day; *pre‐pregnancy*: LMP‐90 days to LMP‐1 day; *first trimester*: LMP to LMP + 97 days; *second trimester*: LMP + 98 days to LMP + 202 days; *third trimester*: LMP + 203 days to delivery‐1 day; *postpartum*: delivery to delivery + 90 days.

Categories of maternal age at delivery were: < 20, 20–24, 25–29, 30–34, 35–39, 40–44, and ≥ 45 years.

### 2.5 Data Analysis

#### 2.5.1 Overall Medication Use

The number and proportion of women with ≥ 1 dispensed prescription during *pregnancy* and *postpartum* were calculated. For the *pregnancy* period, the results were categorized by year and age. For each pregnancy, the number of unique full‐level ATC code dispensed prescriptions during *pregnancy* was calculated, and the number and proportion of pregnancies with 1, 2, 3, 4, 5–9, 10–19, and ≥ 20 unique full‐level ATC code prescriptions were described by year and age.

#### 2.5.2 Most Commonly Used Medications

The 20 most commonly used medications during *pregnancy* and *postpartum* were identified by ranking the proportion of pregnancies with ≥ 1 dispensed prescription in each of the pregnancy periods by full‐level ATC code. To further investigate the patterns of use in and around pregnancy of these 20 most commonly used medications in *pregnancy*, we calculated the number and proportion of women with ≥ 1 dispensed prescription *pre‐pregnancy*, in each *trimester*, and *postpartum*.

To assess which medications were most frequent during each period, the 20 most commonly used medications *pre‐pregnancy* and *trimester 1–3*, separately, were identified. Lastly, the 20 most commonly used medications during *pregnancy* were reported by category of maternal age.

#### 2.5.3 First Trimester Use of Recognized or Suspected Structural Teratogenic Medications

Overall and by year, the number and proportion of women with ≥ 1 dispensed prescription of a recognized or suspected structural teratogenic medication (Table S2) during the first trimester was calculated. Only medications dispensed to ≥ 0.01% of the study population were presented.

#### 2.5.4 Use of Specific Medication Groups of Clinical Interest During Pregnancy and Postpartum

By year, the proportion of women with ≥ 1 dispensed prescription in specific groups of medications of clinical interest (Table S3) during *pregnancy* or *postpartum* was calculated, including: analgesics, anti‐inflammatory medications, anti‐infectives, anticoagulants, antidiabetics, antiemetics, antihypertensives, asthma medications, prescribed supplements, and psychotropics.

Data analysis was conducted using SAS version 9.4 and result visualization using Stata version 15.1.

## 3 Results

### 3.1 Study Population

There were 1 761 183 eligible pregnancies recorded in the Medical Birth Register from 2007 to 2022. At delivery, the majority of women were aged 25–29 years (*n* = 530 512; 30.1%) and 30–34 years (*n* = 617 301; 35.1%). Only 19 976 (1.1%) women were in the lowest (< 20 years) and 4432 (0.3%) in the highest (≥ 45 years) age categories.

### 3.2 Overall Medication Use

**FIGURE 1:**
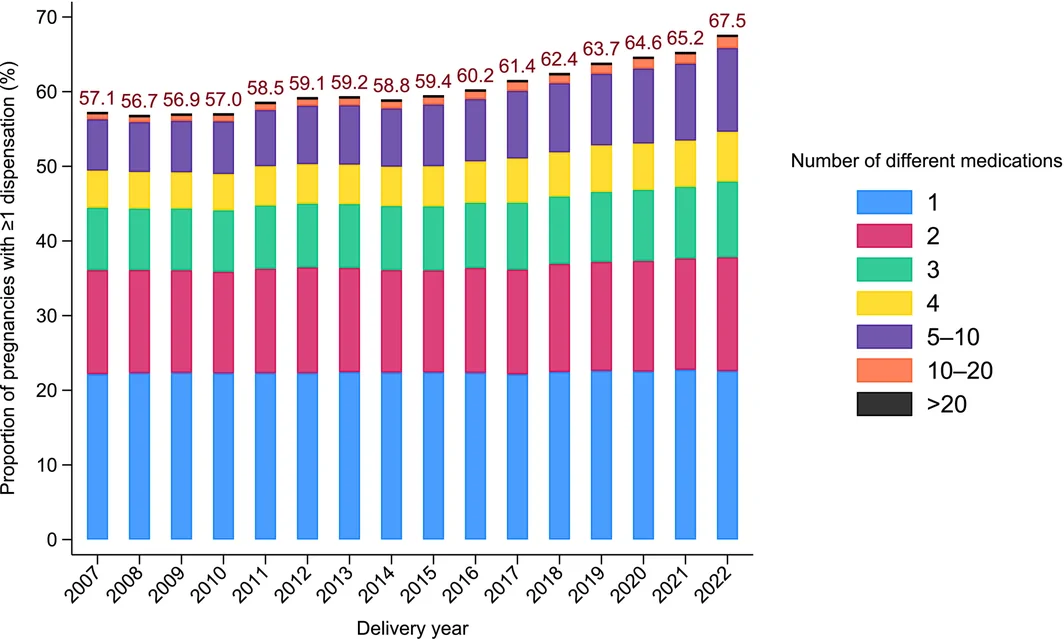
Proportion of pregnancies with at least one dispensed prescription medication during pregnancy per delivery year, stratified by the number of different medications (i.e., unique full‐level ATC codes) dispensed.

In total, 1 065 593 (60.5%) women had a dispensed prescription during *pregnancy*, and 900 536 (51.1%) within 3 months *postpartum*. The proportion during *pregnancy* increased during the study period, from 57.1% in 2007 to 67.5% in 2022 (Figure 1), and the proportion *postpartum* increased from 50.8% in 2007 to 53.5% in 2022. While the proportion of women who were dispensed only one or two different medications during *pregnancy* remained steady during the study period, the use of 5 or more different medications increased over time: 7.7% in 2007 to 12.9% in 2022 (Figure 1).

**FIGURE 2:**
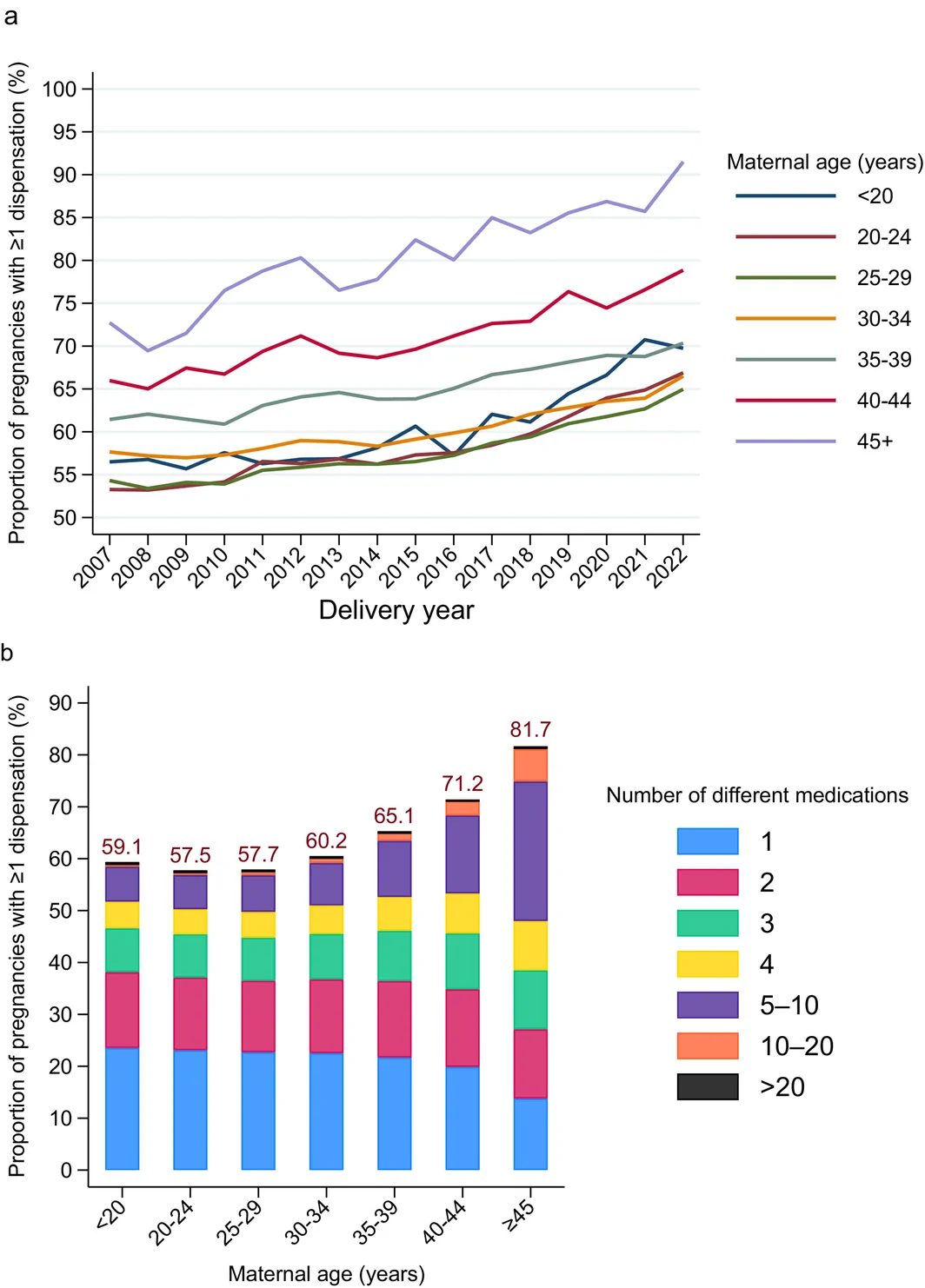
Proportion of pregnancies with at least one dispensed prescription medication during pregnancy by category of maternal age, (a) overall per delivery year, and (b) during the entire study period, stratified on the number of different medications (i.e., unique full‐level ATC codes) dispensed.

Among women in the highest age groups (40–44 years and ≥ 45 years), 71.2% and 81.7%, respectively, had a dispensed prescription during *pregnancy*, compared to 59.1% and 57.5% in the two lowest age groups (< 20 years and 20–24 years), respectively. Between 2007 and 2022, any medication use increased by 14%–26% in all age groups, with the largest increases seen among the youngest (< 20 and 20–24) and oldest (≥ 45 years) women (Figure 2a). The use of several different medications increased with age (Figure 2b). Among 20–24‐year‐olds, 7.3% had five or more different medications dispensed during *pregnancy*, compared to 33.7% of ≥ 45‐year‐olds.

### 3.3 The 20 Most Commonly Dispensed Prescription Medications

**FIGURE 3:**
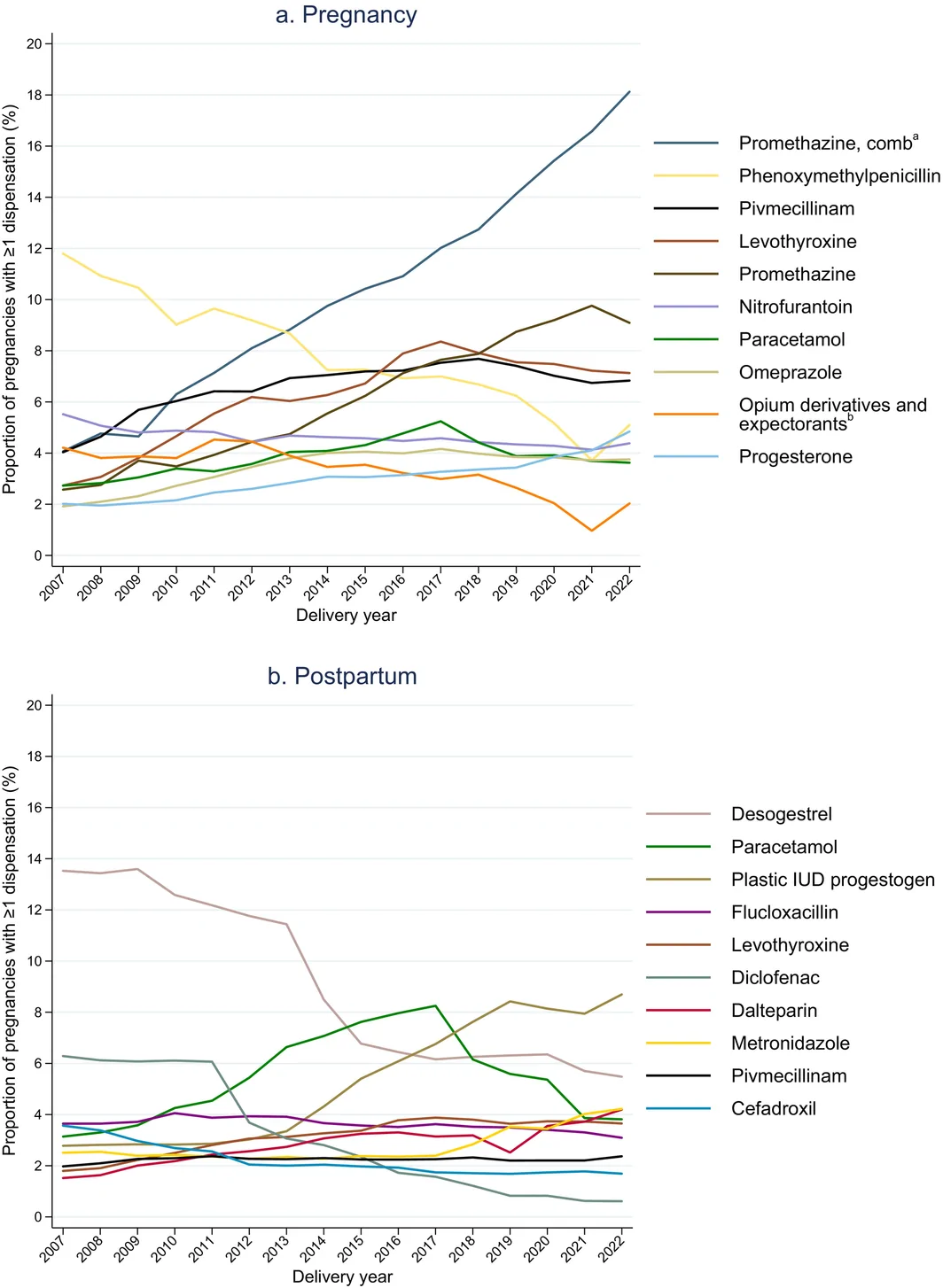
Top 10 most commonly dispensed prescription medications during pregnancy (a) and postpartum (b) in Sweden 2007–2022. Displayed are the proportions of all pregnancies per delivery year with at least one dispensed prescription of each medication. ^a^Promethazine combined with ephedrine and caffeine. ^b^ATC R05FA02, cough syrup containing ethyl morphine, cocillana and senega. Comb., combination; IUD, Intrauterine device.

The most common medications dispensed during *pregnancy* were promethazine and levothyroxine sodium that increased over the study period, and antibiotics, which decreased (Table 1, Figure 3a). *Postpartum*, desogestrel was most common (9.1%) with decreasing use, while other hormonal contraceptives increased (Table 1, Figure 3b). Of the 20 most commonly dispensed medications, 40% (*pregnancy*) and 65% (*postpartum*) were classified as Class 1 in *Janusmed fosterpåverkan* and *Janusmed Amning*, respectively, with the remainder being in Class 2. None were among Class 3 (Table S1).

**TABLE 1:** Top 20 most commonly dispensed prescription medications during pregnancy and postpartum in Sweden 2007–2022, color‐coded according to Swedish pregnancy and breastfeeding medication safety classifications^a^.

	During pregnancy		Postpartum	
	ATC, chemical substance	*n* (%)	ATC, chemical substance	*n* (%)
1	R06AD52, Promethazine combined with ephedrine and caffeine	181 274 (10.3)	G03AC09, Desogestrel	160 283 (9.1)
2	J01CE02, Phenoxymethylpenicillin	137 049 (7.8)	N02BE01, Paracetamol	96 046 (5.5)
3	J01CA08, Pivmecillinam	115 855 (6.6)	G02BA03, Plastic IUD with progestogen	92 818 (5.3)
4	H03AA01, Levothyroxine sodium	109 217 (6.2)	J01CF05, Flucloxacillin	63 856 (3.6)
5	R06AD02, Promethazine	107 229 (6.1)	H03AA01, Levothyroxine sodium	55 623 (3.2)
6	J01XE01, Nitrofurantoin	81 405 (4.6)	M01AB05, Diclofenac	54 380 (3.1)
7	N02BE01, Paracetamol	67 285 (3.8)	B01AB04, Dalteparin	49 724 (2.8)
8	A02BC01, Omeprazole	60 538 (3.4)	P01AB01, Metronidazole	48 721 (2.8)
9	R05FA02, Opium derivatives and expectorants^b^	57 814 (3.3)	J01CA08, Pivmecillinam	39 521 (2.2)
10	G03DA04, Progesterone	53 178 (3.0)	J01DB05, Cefadroxil	38 872 (2.2)
11	B01AC06, Acetylsalicylic acid	53 060 (3.0)	J01CE02, Phenoxymethylpenicillin	35 881 (2.0)
12	B03BA01, Cyanocobalamin	51 548 (2.9)	H01BB02, Oxytocin	33 271 (1.9)
13	N02AJ06, Codeine combined with paracetamol	50 908 (2.9)	M01AE02, Naproxen	32 560 (1.8)
14	R06AA04, Clemastine	50 536 (2.9)	J01CR02, Amoxicillin/Clavulanic acid	32 364 (1.8)
15	B03BB01, Folic acid	48 457 (2.8)	G03AC08, Etonogestrel	30 953 (1.8)
16	R05CB10, Bromhexine combined with ephedrine	45 435 (2.6)	N06AB06, Sertraline	29 008 (1.6)
17	R01AD09, Mometasone (intranasal)	44 231 (2.5)	G03AA07, Levonorgestrel/Ethinylestradiol	21 549 (1.2)
18	R01BA01, Phenylpropanolamine	40 197 (2.3)	N02AJ06, Codeine combined with paracetamol	20 756 (1.2)
19	N06AB06, Sertraline	36 563 (2.1)	J02AC01, Fluconazole	20 209 (1.1)
20	A03FA01, Metoclopramide	33 597 (1.9)	B01AB10, Tinzaparin	20 115 (1.1)

*Note:* Displayed as number and proportion of the total number of pregnancies in the study population (*n* = 1 761 183) with at least one dispensed medication in pregnancy or the 3 months postpartum.

^a^ Classification by the Swedish clinical decision support systems *Janusmed fosterpåverkan* (“Drugs and Fetal Effects”) and *Janusmed Amning* (“Breastfeeding”), respectively. Green = Class 1, includes medications with robust evidence of safety throughout pregnancy or during breastfeeding; Yellow = Class 2, includes medications with either a lack of data or with evidence of mild‐to‐moderate risks associated with specific windows of exposure; Red = Class 3, medications may result in considerable risk to the fetus or breastfed infant. Note that none of the medications listed in this table are included in Class 3. For further information on the classification systems, see the Methods Section and (Table S1).

^b^ A cough syrup containing ethyl morphine, cocillana, and senega.

Of the top 20 medications used during *pregnancy*, some were primarily dispensed during the *first trimester* (e.g., promethazine) or during *pre‐pregnancy* and the *first trimester* (e.g., progesterone), while others had a stable proportion of women across all *trimesters* (e.g., omeprazole, phenoxymethylpenicillin, and sertraline; Table S4). Table S5 displays the top 20 medications separately for the *pre‐pregnancy* period and *each trimester*.

The most common dispensed medications during *pregnancy* were generally consistent across maternal age categories. The proportion of women with episodic medications such as antiemetics and antibiotics was similar across age groups, while medications such as hormones, acetylsalicylic acid, anticoagulants, and antihypertensives were more commonly used in women with higher age (Table S6).

### 3.4 First Trimester Use of Recognized or Suspected Structural Teratogenic Medications

**FIGURE 4:**
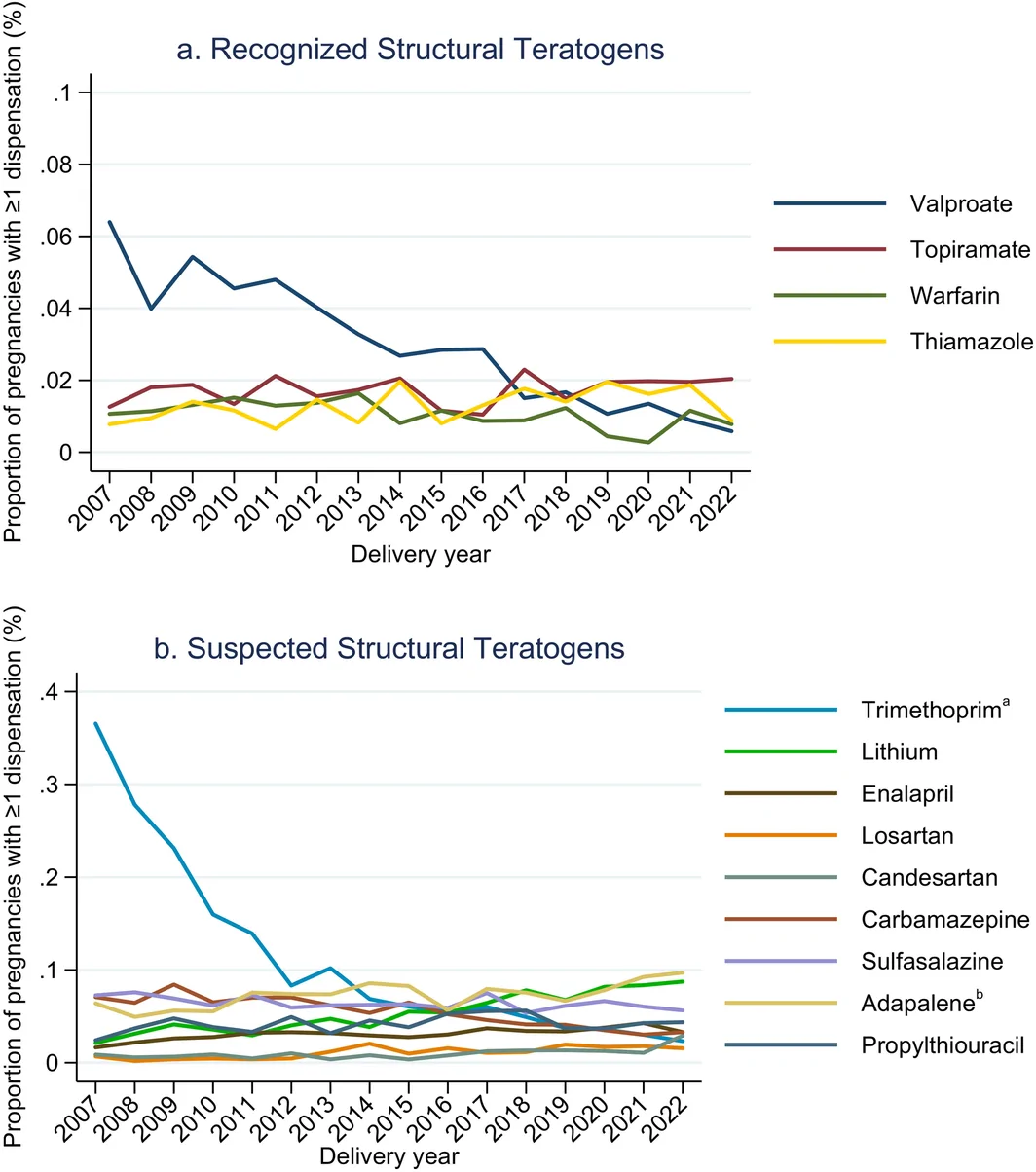
Proportion of pregnancies in Sweden 2007–2022 with at least one first trimester dispensed prescription of selected recognized (a) and suspected (b) structural teratogenic medications. Medications dispensed by less than 0.01% of the study population are not displayed. ^a^Trimethoprim alone or in combination with sulfamethoxazole. ^b^Topical formulation.

The proportion of pregnant women dispensed recognized (1686; 0.10%) or suspected (8499; 0.48%) structural teratogens in the *first trimester* was low and stable across the study period (Figure 4a,b). The most common recognized and suspected structural teratogens, valproate and trimethoprim respectively, decreased markedly.

### 3.5 Use of Specific Medication Groups of Clinical Interest

**FIGURE 5:**
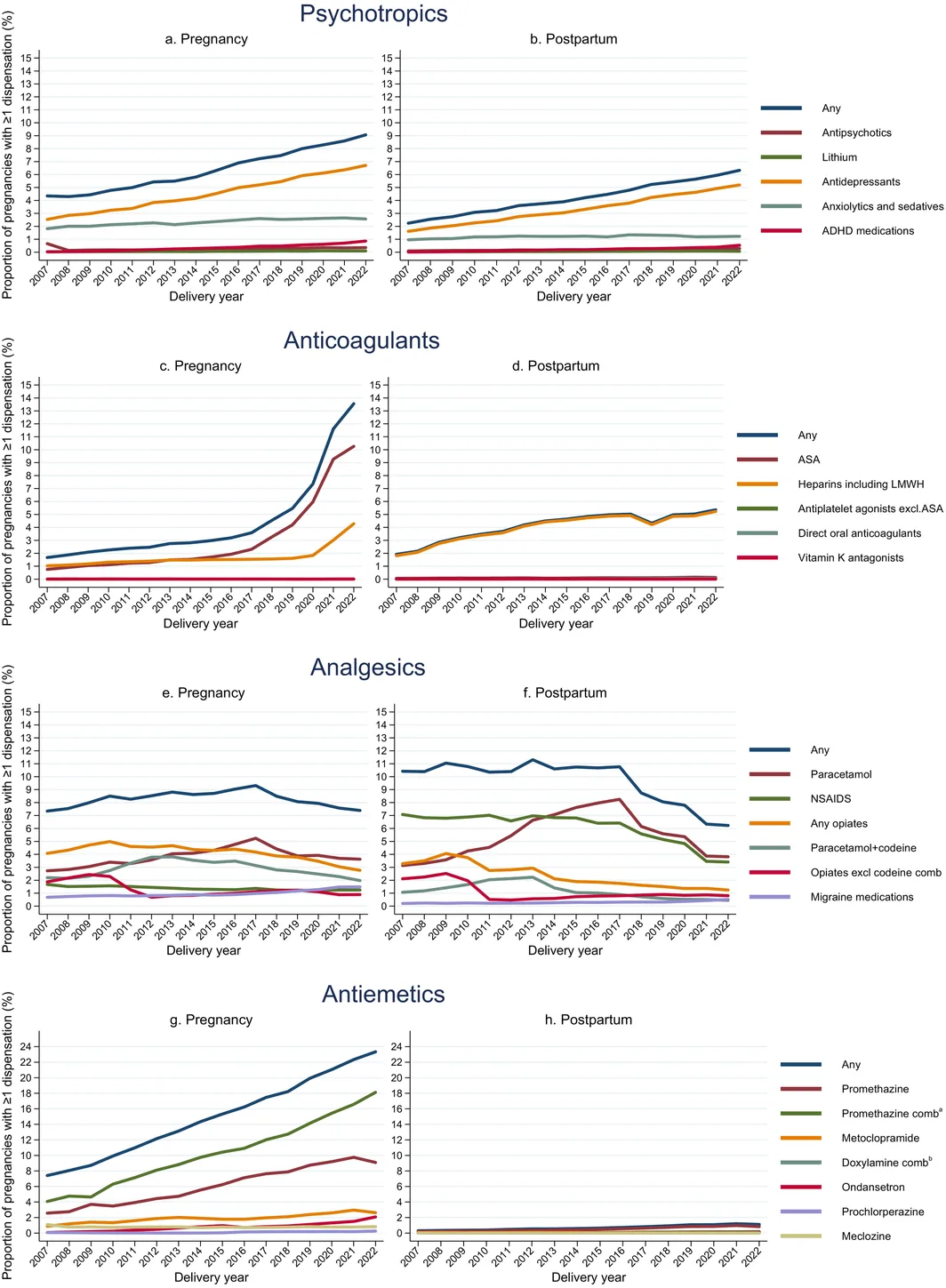
Proportion of pregnancies in Sweden 2007–2022 with at least one dispensed prescription medication within specific medication groups of interest during pregnancy (left) and in the 3 months postpartum (right) for psychotropics (a and b), anticoagulants (c and d), analgesics (e and f), and antiemetics (g and h). ASA, Acetylsalicylic acid; comb., combination; LMWH, low molecular weight heparin; NSAIDS, non‐steroidal anti‐inflammatory drugs. Medication groups defined in Table S3. The data sources used for this study do not capture over‐the‐counter medication or medication administered in hospital. ^a^Promethazine combined with ephedrine and caffeine. ^b^Doxylamine combined with pyridoxin (vitamin B6).

From 2007 to 2022, the proportion of women dispensed antiemetics, acetylsalicylic acid, antidepressants, and metformin increased more than 2‐fold during *pregnancy* (Figures 5 and S1). The proportion of women dispensed antihypertensive medications was similar during both *pregnancy* and *postpartum*, increasing from around 1.5% to 4%–5% (Figure S1a,b). The proportion of women dispensed analgesics during *pregnancy* was stable, but *postpartum* there was a decrease from 10.4% to 6.2%, mainly driven by a decreased proportion of women who filled prescriptions of paracetamol and non‐steroidal anti‐inflammatories (NSAIDs) (Figures 3b, 5f). During both pregnancy and postpartum, the proportion of women with dispensed prescriptions of opiate‐containing medication except codeine decreased by half, from 2% at the start of the study period (2007–2009) to 1% from 2012 onward (Figure 5e,f). Further results on the specific medication groups of interest are presented in Supporting Information Results.

## 4 Discussion

The last comprehensive assessment of medication use in pregnant women in Sweden was based on data from the first complete year (2007) of the Prescribed Drug Register [6, 33]. Over the subsequent 15‐year study period between 2007 and 2022, the proportion of pregnant women using at least one prescribed medication increased from 57% to 68%, as did the proportion using five or more medications, with the largest increase among women of higher age. The most common medications used in pregnancy included antibiotics, antiemetics, and levothyroxine sodium. In the postpartum period, medication use remained steady over the study period (51%–54%), and the most common medications were contraceptives, antibiotics, and analgesics. The proportion of women with recognized and suspected structural teratogens in the first trimester remained low. Use of antiemetics, antidiabetics, psychotropics, antihypertensives, and anticoagulants during pregnancy increased, whereas the use of antibiotics, non‐steroidal anti‐inflammatory medications (NSAIDs), and opiates decreased.

Similar trends of medication use among pregnant women have been reported in Denmark, a country with a comparable population, healthcare systems, and national register data. Thunbo et al. [3] reported that in Denmark, dispensation of at least one prescription medication during pregnancy increased from 56.9% in 1998 to 63.3% in 2018, with a larger increase in the youngest and the oldest age groups, and an increase in the use of multiple medications, particularly in women with higher age. In contrast, a study based on insurance‐claims data from 2015 to 2021 in Switzerland reported that 87.5% of the study population dispensed at least one medication during pregnancy, with 33% of pregnant women dispensing 5 or more medications [2], while our findings in Sweden were 68% and 13% in 2022, respectively. While the age distribution was similar in both Nordic and Swiss cohorts, prescription traditions, over‐the‐counter availability, and reimbursement policies may underlie these differences.

Our findings are reflective of changing maternal demographics including older age and obesity, more comorbidities, higher prevalence of pregnancy complications and assisted reproduction use, as well as improved adherence to management recommendations during pregnancy [8, 11, 12, 13]. Further, new effective treatments have become available for conditions, such as inflammatory and autoimmune disorders, allowing for improved disease control and leading to more women with these conditions pursuing pregnancy [40, 41]. Information about the indication of medication use is not available in the national register data, however the indication, and whether it was present before pregnancy or has its onset during or after pregnancy directly influences the observed pattern of medication use over the pregnancy periods. For many of the most commonly dispensed medications, use was higher during pregnancy than before, with several of these patterns plausibly reflecting pregnancy‐specific indications. For example, promethazine is primarily dispensed in the first trimester which corresponds well with its role as one of the first‐line antiemetics in Sweden [42], and a higher proportion of progesterone (specifically ATC code G03DA04) users during pregnancy reflects its use for luteal phase support as part of an assisted reproduction treatment program and for cervical insufficiency. Whereas, medications for which there is a stable proportion of women dispensing them over the pregnancy periods (e.g., omeprazole, phenoxymethylpenicillin, and sertraline) the patterns of use are more difficult to interpret without information on the number of incident or prevalent users. Further, while overall the proportion of women using medication postpartum was lower compared to during pregnancy, an exception is the use of prescribed analgesics, their higher postpartum use may reflect the management of postpartum pain, including pain associated with perineal trauma, post‐operative recovery after cesarean section, or breastfeeding complications such as mastitis.

Our results are in line with an American study showing that the most commonly reported medications were antibiotics, antiemetics, and analgesics [1]. While antibiotics remain one of the most prescribed medications both during pregnancy and the postpartum period, the decline in use observed in our data is consistent with global trends, largely attributable to the work of organizations promoting rational antibiotic use [43, 44, 45]. Although, the prevalence of antiemetics was fairly similar, the type of medications differed between the two cohorts. The most commonly used antiemetic in the US was ondansetron, followed by doxylamine/pyridoxin, promethazine, and metoclopramide, compared to promethazine being the most prescribed antiemetic during pregnancy in Sweden, which follows current treatment recommendations [42].

The proportion of pregnant women using recognized structural teratogens in the first trimester was very low and stable over the study period, compared to other cohorts, which could be due to differing definitions of teratogenic drugs as well as characteristics of the study populations [1, 4, 37, 38, 39]. We defined recognized structural teratogens as medications with proof of teratogenicity based on human data, or strong evidence from animal data. Suspected teratogens were medications with some indications of teratogenicity from human data, but with lower or conflicting risk estimates. The most dispensed recognized structural teratogen was valproate (0.03%), with use lower than previously reported, with further decreasing use over time. This is likely due to the increased knowledge and warnings regarding teratogenicity as well as evidence on the safety of other antiseizure and psychotropic medications [14, 46, 47, 48, 49, 50, 51]. The most dispensed suspected structural teratogen was trimethoprim, with a decreased use from 0.35% to 0.03%. Trimethoprim is an antagonist of folic acid, but its teratogenic potential has not been confirmed [52, 53]. In general, our findings regarding low use of structural teratogens are reassuring, and could in part be attributed to the well‐developed Swedish knowledge base for drug safety during pregnancy, *Janusmed fosterpåverkan*, which is easily accessible and widely used by prescribers, pharmacies and the public [54].

During pregnancy, we observed increasing use of medications indicated for high‐risk pregnancies such as anticoagulants, antihypertensives, and hormones, especially in older aged women, consistent with a rising prevalence of pregnancy‐related disorders [55, 56, 57]. In particular, the parallel increase in acetylsalicylic acid and antihypertensives may reflect increased efforts in managing hypertensive disorders of pregnancy [13]. We also noted an increase in antidiabetic medication use from 2015 onward, which may be due to changes in diagnostic criteria for gestational diabetes as well as the growing acceptance of metformin as an appropriate treatment during pregnancy and is in line with trends of antidiabetics elsewhere in the world [58]. These patterns of medication use may not only indicate an increase in disease onset during pregnancy itself but may also reflect changes in the underlying prevalence of chronic conditions [8]. The rise in use of antidepressants during pregnancy and postpartum in this study is in line with prescription trends internationally [59, 60, 61], while the use of antipsychotics was steady and much lower than what is seen in other parts of the world [62]. The use of opioids was low and decreased, similar to other settings, although opioid use during pregnancy in general largely differs between countries and socioeconomic levels [63]. The use of lithium was low but increased, in line with the other Nordic countries [64].

Postpartum, we observed a shift in the types of contraceptives used, reflective of changes in clinical practice with hormonal intrauterine devices recommended as the preferred form of contraception immediately postpartum [65]. In contrast, most other postpartum medications remained stable from 2007 to 2022, suggesting consistency in prescribing practices. An exception was diclofenac, for which we observed a gradual decline, potentially reflecting increased caution regarding its safety profile in comparison with other NSAIDs and growing awareness of its environmental impact [66]. Overall, the stability of postpartum medication use is reassuring in the context of breastfeeding, as it may reflect continued reliance on medications with well‐established safety profiles for lactating individuals. However, like most other studies, we lacked information on breastfeeding [30].

### 4.1 Strengths and Limitations

A major strength of this study is the use of population‐based data that are prospectively collected during routine healthcare. This minimizes recall bias and ensures near‐complete coverage [31]. Additionally, the Prescribed Drug Register captures information on all medications dispensed from outpatient pharmacies, including prescriptions issued in both public and private primary and specialist care.

Some limitations should be considered. First, the Prescribed Drug Register does not include medications administered in specialist outpatient settings, such as intravenous antirheumatic or antineoplastic medications which are expected to be rare in pregnancy, or medications given during hospitalization. While this latter limitation means that acute short‐term medication use during hospitalization was not captured, medication initiated in‐hospital and then prescribed for use after discharge was. Additionally, the Prescribed Drug Register does not include data on medications sold over‐the‐counter, such as analgesics, antacids, and vitamins nor medications acquired outside the regulated prescription and pharmacy dispensing systems. As a result, the use of certain common medications, such as paracetamol, is underestimated because they are often recommended for purchase without a prescription in Sweden.

Second, we do not know if women dispensed a medication have actually taken it. While the Medical Birth Register contains information on maternal self‐reported medication use in early pregnancy, these data were not included in the present study as we aimed to provide a comprehensive overview of dispensed prescriptions also before pregnancy, throughout each trimester, and postpartum. Further, information on the indications for medication use, the duration of medication use, or whether the medication was intended for chronic or as‐needed use is not readily available. While proxies based on diagnoses codes and dispensed supply may be used to approximate indication and estimate duration, these approaches should be medication specific, and therefore not within the scope of this overview study.

Third, due to register coverage, medication use for pregnancies ending before 22 weeks of gestation is not captured.

## 5 Conclusion

Medication use during pregnancy in Sweden increased between 2007 and 2022. The described trends underscore the importance of ongoing surveillance and further research on safety during pregnancy and breastfeeding, particularly for medication groups with increasing use and limited available evidence.

## Author Contributions

C.E.C., P.K., E.W.H., A.I.S. conceptualized the study. All authors contributed to the design of the study. C.E.C. acquired the data. P.K. performed the analyses. E.W.H., A.I.S., C.E.C., S.S.C. wrote the initial draft. All authors interpreted the data, contributed to the adjustment of the manuscript, provided intellectual content, and approved the final version of the manuscript. C.E.C. is the guarantor of this work and, as such, had full access to all the data in the study and takes responsibility for the integrity of the data and the accuracy of the data analysis.

## Funding

E.W.H. was supported by the Freemasons Children's Welfare Foundation in Stockholm. A.I.S. was supported by a clinical postdoc appointment from Region Stockholm. A.K.Ö. was supported by a grant from the Swedish Research Council for Health, Working Life and Welfare (no. 2024‐00420). C.A. was supported by grants from the Swedish Research Council (no. 2024‐02626) and the Swedish Research Council for Health, Working Life and Welfare (no. 2024‐00806). C.E.C. was supported by a grant from the Swedish Research Council (no. 2024‐02749). The funders played no role in design, data analyses, or interpretation of findings; writing the report or the decision to submit the manuscript for publication.

## Disclosure

Data transparency and reproducibility: The manuscript is aligned with guidance from the reporting checklist RECORD‐PE [67]. The checklist is provided in the Supporting Information. The study, which does not include any hypothesis testing, was not preregistered and the protocol is not available. The authors are not permitted to directly share the Swedish national health register data; access can be requested from the relevant Swedish register holders after approval by the Swedish Ethical Review Board. The analytical code is available upon request.

## Ethics Statement

This study is covered under the following approval and amendments from the Swedish Ethical Review Board: 2015/1826‐31/2; 2017/2238‐32; 2018/1790‐32; 2018/2211‐32; 2022‐04004‐02; 2023‐01622‐02.

## Conflicts of Interest

C.E.C., P.K., and S.S.C. report participation in regulator‐mandated Phase IV studies funded by pharmaceutical companies, all with funds paid to the institution where they are employed, and with no relation to the work reported in this paper. C.A. reports that her spouse is employed by a company (Bristol Myers Squibb) whose products are included among those being studied; C.A. had no interaction with the company and received no funding or input from the company. The other authors declare no conflicts of interest.

## Supporting information


**Table S1:** (A and B): Medication classifications* in *Janusmed fosterpåverkan* (fetal effects) and *Janusmed amning* (breastfeeding).
**Table S2:** ATC codes for recognized and suspected structural teratogens.
**Table S3:** ATC codes for the specific medication groups of interest.
**Table S4:** Top 20 most commonly dispensed prescription medications overall during pregnancy in Sweden 2007–2022 with corresponding proportions in each pregnancy period*.
**Table S5:** Top 20 most commonly dispensed prescription medications pre‐pregnancy and in each trimester in Sweden 2007–2022*.
**Table S6:** Top 20 most commonly dispensed prescription medications during pregnancy in Sweden 2007–2022 by category of maternal age at delivery.
**Figure S1:** (a–f) Proportion of pregnancies in Sweden 2007–2022 with at least one dispensed prescription within specific medication groups of interest during pregnancy (left) and in the 3 months postpartum (right) for antihypertensives (a and b), antidiabetics (c and d), antirheumatic medications (e and f). Medication groups defined in Table S3.
**Figure S2:** (a–f) Proportion of pregnancies in Sweden 2007–2022 with at least one dispensed prescription within specific medication groups of interest during pregnancy (left) and in the 3 months postpartum (right) for anti‐infectives (a and b), asthma medications (c and d) and prescribed supplements (e and f). Medication groups defined in Table S3.

**Data S1:** pds70473‐sup‐0002‐Supinfo.pdf.

## Data Availability

The authors are not permitted to directly share the Swedish national health register data; access can be requested from the relevant Swedish register holders after approval by the Swedish Ethical Review Board. The analytical code is available upon request.
