# Supplementary material for: Prescription Medication Use During Pregnancy and Postpartum in Sweden 2007 to 2022
Source: Pharmacoepidemiol Drug Saf. 2026 Sep 9;35(9):e70473. doi: 10.1002/pds.70473 (PMC13557928; doi:10.1002/pds.70473)
Supplement: Supplementary file 2 [file PDS-35-e70473-s002.pdf]

Supplementary Material and Results

Prescription Medication Use During Pregnancy and Postpartum in Sweden 2007 to 2022

Essi Whaites Heinonen, Awad I. Smew, Silvia Segovia Chacón, Ulrika Nörby, Olof Stephansson, Anne K. Örtqvist, Cathrine Axfors, Pär Karlsson, Carolyn E. Cesta*

*Corresponding author: carolyn.cesta@ki.se

Table of Contents

Supplementary Materials **2**

Supplementary Table 1 a and b: Medication Classifications* in *Janusmed fosterpåverkan* (fetal effects) and *Janusmed amning* (breastfeeding) 2

Supplementary Table 2: ATC codes for Recognized and Suspected Structural Teratogens 3

Supplementary Table 3. ATC codes for the specific medication groups of interest 5

Supplementary Results **7**

Supplementary Table 4. Top 20 most commonly dispensed prescription medications overall during pregnancy in Sweden 2007–2022 with corresponding proportions in each pregnancy period* 7

Supplementary Table 5. Top 20 most commonly dispensed prescription medications pre-pregnancy and in each trimester in Sweden 2007–2022* 8

Supplementary Table 6. Top 20 most commonly dispensed prescription medications during pregnancy in Sweden 2007–2022 by category of maternal age at delivery 10

Supplementary Figure 1 A-F 12

Supplementary Figure 2 A-F 13

Supplementary description of results for specific medication groups of interest during pregnancy and in the three months postpartum 14

Supplementary Materials

Supplementary Table 1 a and b: Medication Classifications* in *Janusmed fosterpåverkan* (fetal effects) and *Janusmed amning* (breastfeeding)

A

**Class 1**	**The drug may be used during pregnancy without any increased fetal risk**
**Class 2**	**Important information is available about this drug during pregnancy**<br>The experience of the drug may be limited. Therefore, the drug should be used with care. The drug may also be suspected of causing – or in rare cases have been demonstrated to cause – a moderate increase in the risk of teratogenic or other negative effects on the fetus.
**Class 3**	**If used during pregnancy, the drug will entail or may be suspected of entailing a considerable risk to the fetus**<br>The patient should be informed about the risk to the expected fetus. Exposure should result in a discussion about how the pregnancy should continue to be managed.

B

**Class 1**	**Compatible with breastfeeding**
**Class 2**	**Compatible with breastfeeding only under certain circumstances**
**Class 3**	**Use not recommended during breastfeeding**

*****A) Official English translation of Swedish text found at https://janusmed.se/about/omfosterpaverkan on October 23, 2025. B) Translated from the original medication classification tables found in Swedish at https://janusmed.se/about/omamning on Oct 23, 2025.

Note: These classifications provide a simplified picture, and the users of *Janusmed fosterpåverkan* and *Janusmed Amning* are encouraged to read the contextual information in narrative format available for each medication to obtain a more complete risk assessment.

Supplementary Table 2: ATC codes for Recognized and Suspected Structural Teratogens

Category	Medication ATC subgroup or substance name	ATC code
**Recognized structural teratogens**		
Synthetic prostaglandins	misoprostol, and other syntetic prostaglandins	M01AB55, A02BB01,** G02AD06,* M01AE56,* G03XB01,** G03XB51,* G02AD01-04*
Vitamin K agonists	warfarin, fenprocoumon, acenocoumarol	B01AA03, B01AA04,** B01AA07*
Antihypertensives for pulmonary arterial hypertension	antihypertensives for pulmonary arterial hypertension	C02KX**
Vasopressin antagonist	tolvaptan	C03XA01,* G04BX21*
Systemic retinoids	etretinate, acitretin, isotretinoin, alitretinoin	D05BB,** D10BA01,** D11AH04*
Androgens	androgens	G03B,** G03E*
Antigonadotropins and similar agents	danazol	G03XA01*
Antineoplastic medications	antineoplastic medications	L01AA02,** L01BB03,** L01BC02,** L01EA03,** L01EA01-3,** L01EA05,** L01EJ01,** All other L01 except L01BB02 (that is not considered a teratogen)*
Anti-androgens	anti-androgens	L02BB**, G03HA01*
Immunosuppressive medications	mycophenolic acid, leflunomide, fingolimod, tofacitinib, teriflunomide, baricitinib, ozanimod, cladribine, siponimod, sirolimus,thalidomide, methotrexate, lenalidomide, pomalidomide	L04AA06,** L04AE01,** L04AF01,** L04AF02,** L04AX03,** L04AH01,* L04AK01,* L04AK02,* L04AE02,* L04AA40,* L04AE03,* L04AX02,* L04AX04,* L04AX06*
Vascular endothelin-like growth factor-A inhibitors	ranibizumab, aflibercept	S01LA04-05*
Teratogenic antithyroid agents	thiamazole	H03BB02
Teratogenic antiseizure medications	valproic acid, topiramate	N03AG01, N03AX11
Podophyllotoxin derivatives	Podophyllotoxin derivatives not included in L01	M01C*
Antiestrogens	tamoxifen, toremifem, fulvestrant, elacestrant	L02BA01,** L02BA02-04*
Testosterone 5-alpha-reductase inhibitors	finasteride, dutasteride	D11AX10,* G04CB02*
**Suspected Structural Teratogens**		
Medication acting on the renin-angiotensin-aldosterone system	ACE-inhibitors, ARBs, other RAAS-acting drugs	C09AA02, C09CA01, C09CA06, C09AA01,** , C09AA03,** C09AA05,** C09AA08,** C09BA02,** C09BA05,** C09CA02,** C09CA04,** C09CA07,** C09DA01,** C09DA03-04,** C09DA06-07,** C09DB01,** All other C09*
Retinoids for topical use in acne	topical adapalene, isotretinoin and tretinoin	D10AD03, D10AD53, D10AD01,** D10AD51,** D10AD04,* D10AD54*

Anti-thyroid agents	propylthiouracil	H03BA02
Folic acid antagonists	trimethoprim, trimethoprim with sulfonamides, sulfasalazine	J01EA01, J01EE, A07EC01
Other immunosuppressants	penicillamine	M01CC01*
Agents used in gout and hyperuricaemia	colchicine	M04AC01**
Selected antiseizure medications with suspected structural teratogenic potential	carbamazepine, oxcarbaxepine phenobarbital, primidone, fenytoin, ethosuximide, oxcarbazepine, eliscarbazepine, vigabatrine, zonisamide, lacosamide, perampanel, bivaracetam	N03AF01, N03AA02-03,** N03AB02,** N03AD01,** N03AF02,** N03AF04,** N03AG04,** N03AX15,** N03AX18,** N03AX22-23
Antivirals with suspected teratogenic potential	ribavirin, ganciclovir, cidofovir, valganciclovir, foscarnet, idoxuridine	J05AB02,* J05AB04,* J05AB06,* J05AB12,* J05AB14,* J05AD01,* J05AP01*
Antipsychotic	lithium	N05AN, N05AN01

Abbreviations: ATC= anatomic therapeutic classification, ACE=Angiotensin converting enzyme, ARB=angiotensin 2 receptor blockers, RAAS=renin-angiotensin-aldosterone system

*no pregnancies in the study population with a dispensed prescription of the particular ATC substance during the first trimester

** <0.01% of pregnancies in the study population with a dispensed prescription of the specific ATC substance during the first trimester

Supplementary Table 3. ATC codes for the specific medication groups of interest

Medications	ATC-codes
**Psychotropics**	N05, N06
Antipsychotics	N05AA-N05AB03, N05AB05-N05AL, N05AX
Lithium	N05AN
Antidepressants	N06A
Anxiolytics, hypnotics and sedatives	N05B, N05C
ADHD-medications	N06BA, C02AC02
**Anticoagulants**	B01A
ASA	B01AC06
Vitamin K antagonists	B01AA
Heparins including LMWH	B01AB
Antiplatelet agonists except ASA	B01AC-B01AC05, B01AC07-B01AC27
Direct oral anticoagulants	B01AE07, B01AF
**Analgesics**	N02A, N02B, N02C, M01A
Paracetamol	N02BE01
NSAIDS	M01A
Any opiates	N02A
Paracetamol + codein	N02AJ06
Opiates except codeins	N02A-N02AJ05, N02AJ13-N02AJ23
Migraine medications	N02C
**Antiemetics**	R06AD52, R06AA59, A04AA01, N05AB04, R06AD02, A03FA01, R06AE05
Promethazine	R06AD02
Promethazine combination with caffeine and ephedrine	R06AD52
Metoclopramide	A03FA01
Doxylamine combinations	R06AA59
Ondansetron	A04AA01
Prochlorperazine	N05AB04
Meclozine	R06AE05
**Antihypertensives**	C01-C04, C07-C09
RAAS inhibitors	C09
Cardiac medications	C01
Other antihypertensives	C02
Diuretics	C03
Peripheral vasodilators	C04
Beta blockers	C07
Calcium inhibitors	C08
**Antidiabetics**	**A10A, A10B**
Non-insulins	A10B
Insulin	A10A
Metformin	A10BA02
Other glucose lowering drugs except for insulin and metformin	A10B, except A10BA02
**Antirheumatic medications**	H02AB, L04A, L01
Systemic steroids	H02AB

Monoclonal antibodies	L04AG, L04AC01-02, L04AC04-16, L04AB02-05
Etanercept	L04AB01
Azathioprine	L04AX01
Mercaptopurine	L01BB02
Antineoplastics	L01 except L01BB02
**Asthma medications**	R03
**Prescribed supplements**	
Iron	B03AA
Folic Acid	B03BB
D-Vitamin	A11CC
Calcium + D-vitamin	A12AX
Cyanocobalamin	B03BA01
**Anti-infectives**	
Antibiotics	J01
Antivirals	J05

Abbreviations: RAAS, Renin-Angiotensin-Aldosterone System; ADHD,Attention Deficit Hyperactivity Disorder; ASA, Acetylsalicylic acid; LMWH=Low Molecular Weight Heparins; NSAIDS, Non-steroidal Anti-inflammatory Drugs.

Supplementary Results

Supplementary Table 4. Top 20 most commonly dispensed prescription medications overall during pregnancy in Sweden 2007–2022 with corresponding proportions in each pregnancy period*

		n (%)					
	**ATC, chemical substance**	**Pre-pregnancy**	**Top 20 most commonly dispensed medications overall during pregnancy**	**First trimester**	**Second trimester**	**Third trimester**	**Postpartum**
**1**	R06AD52, Promethazine combined with ephedrine and caffeine	1,471 (0.1)	181,274 (10.3)	160,813 (9.1)	61,763 (3.5)	18,224 (1.0)	1,611 (0.1)
**2**	J01CE02, Phenoxymethylpenicillin	44,412 (2.5)	137,049 (7.8)	42,713 (2.4)	58,887 (3.3)	46,387 (2.6)	35,881 (2.0)
**3**	J01CA08, Pivmecillinam	27,627 (1.6)	115,855 (6.6)	45,605 (2.6)	49,485 (2.8)	32,053 (1.8)	39,521 (2.2)
**4**	H03AA01, Levothyroxine sodium	51,858 (2.9)	109,217 (6.2)	85,298 (4.8)	94,284 (5.4)	72,303 (4.1)	55,623 (3.2)
**5**	R06AD02, Promethazine	8,546 (0.5)	107,229 (6.1)	60,996 (3.5)	37,711 (2.1)	37,172 (2.1)	9,332 (0.5)
**6**	J01XE01, Nitrofurantoin	15,747 (0.9)	81,405 (4.6)	26,093 (1.5)	37,230 (2.1)	28,259 (1.6)	8,071 (0.5)
**7**	N02BE01, Paracetamol	34,411 (2.0)	67,285 (3.8)	28,958 (1.6)	33,423 (1.9)	26,791 (1.5)	96,046 (5.5)
**8**	A02BC01, Omeprazole	20,901 (1.2)	60,538 (3.4)	28,943 (1.6)	26,855 (1.5)	23,402 (1.3)	14,549 (0.8)
**9**	R05FA02, Opium derivatives and expectorants**	19,183 (1.1)	57,814 (3.3)	16,674 (0.9)	24,870 (1.4)	18,494 (1.1)	4,151 (0.2)
**10**	B01AC06, Acetylsalicylic acid	46,397 (2.6)	53,178 (3.0)	50,998 (2.9)	3,710 (0.2)	1,751 (0.1)	196 (0.0)
**11**	G03DA04, Progesterone	3,919 (0.2)	53,060 (3.0)	40,573 (2.3)	40,925 (2.3)	13,193 (0.7)	1,725 (0.1)
**12**	B03BA01, Cyanocobalamine	11,654 (0.7)	51,548 (2.9)	15,995 (0.9)	26,776 (1.5)	30,265 (1.7)	11,089 (0.6)
**13**	R06AA04, Clemastine	15,767 (0.9)	50,908 (2.9)	13,857 (0.8)	21,852 (1.2)	25,279 (1.4)	20,756 (1.2)
**14**	N02AJ06, Codeine combined with paracetamol	4,404 (0.3)	50,536 (2.9)	11,178 (0.6)	18,078 (1.0)	26,318 (1.5)	10,344 (0.6)
**15**	B03BB01, Folic acid	16,410 (0.9)	48,457 (2.8)	29,115 (1.7)	21,154 (1.2)	16,561 (0.9)	6,109 (0.3)
**16**	R05CB10, Bromhexine combined with ephedrine	11,324 (0.6)	45,435 (2.6)	11,272 (0.6)	20,288 (1.2)	15,325 (0.9)	4,819 (0.3)
**17**	R01AD09, Mometasone	21,771 (1.2)	44,231 (2.5)	16,181 (0.9)	19,631 (1.1)	14,260 (0.8)	8,734 (0.5)
**18**	R01BA01, Phenylpropanolamine	9,694 (0.6)	40,197 (2.3)	10,740 (0.6)	19,120 (1.1)	13,836 (0.8)	3,943 (0.2)
**19**	N06AB06, Sertraline	27,567 (1.6)	36,563 (2.1)	25,976 (1.5)	25,347 (1.4)	20,490 (1.2)	29,008 (1.6)
**20**	A03FA01, Metoclopramide	2,250 (0.1)	33,597 (1.9)	27,871 (1.6)	9,723 (0.6)	1,636 (0.1)	1,207 (0.1)

*Displayed as number and proportion of the total number of pregnancies in the study population (N=1,761,183) with at least one dispensed prescription in each respective pregnancy period

** A cough syrup containing ethylmorphine, cocillana and senega

Supplementary Table 5. Top 20 most commonly dispensed prescription medications pre-pregnancy and in each trimester in Sweden 2007–2022*

	Pre-pregnancy		*First trimester*		*Second trimester*		*Third trimester*	
	**ATC, chemical substance**	**n (%)**	**ATC, chemical substance**	**n (%)**	**ATC, chemical substance**	**n (%)**	**ATC, chemical substance**	**n (%)**
**1**	H03AA01, Levothyroxine sodium	51,858 (2.9)	R06AD52, Promethazine combined with ephedrine and caffeine	160,813 (9.1)	H03AA01, Levothyroxine sodium	94,284 (5.4)	H03AA01, Levothyroxine sodium	72,303 (4.1)
**2**	G03DA04, Progesterone	46,397 (2.6)	H03AA01, Levothyroxine sodium	85,298 (4.8)	R06AD52, Promethazine combined with ephedrine and caffeine	61,763 (3.5)	J01CE02, Phenoximethyl-penicillin	46,387 (2.6)
**3**	J01CE02, Phenoximethyl-penicillin	44,412 (2.5)	R06AD02, Promethazine	60,996 (3.5)	J01CE02, Phenoximethyl-penicillin	58,887 (3.3)	R06AD02, Promethazine	37,172 (2.1)
**4**	G03GA08, Choriogonadotropin-alfa	41,881 (2.4)	G03DA04, Progesterone	50,998 (2.9)	J01CA08, Pivmecillinam	49,485 (2.8)	J01CA08, Pivmecillinam	32,053 (1.8)
**5**	N02BE01, Paracetamol	34,411 (2.0)	J01CA08, Pivmecillinam	45,605 (2.6)	B01AC06, Acetylsalicylic acid	40,925 (2.3)	B03BA01, Cyanocobalamine	30,265 (1.7)
**6**	J01CA08, Pivmecillinam	27,627 (1.6)	J01CE02, Phenoximethyl-penicillin	42,713 (2.4)	R06AD02, Promethazine	37,711 (2.1)	J01XE01, Nitrofurantoin	28,259 (1.6)
**7**	N06AB06, Sertraline	27,567 (1.6)	B01AC06, Acetylsalicylic acid	40,573 (2.3)	J01XE01, Nitrofurantoin	37,230 (2.1)	N02BE01, Paracetamol	26,791 (1.5)
**8**	G03GA05, Follitropin-alfa	25,087 (1.4)	B03BB01, Folic acid	29,115 (1.7)	N02BE01, Paracetamol	33,423 (1.9)	R06AA04, Clemastine	26,318 (1.5)
**9**	H01CC01, Ganirelix	23,171 (1.3)	N02BE01, Paracetamol	28,958 (1.6)	A02BC01, Omeprazole	26,855 (1.5)	N02AJ06, Codeine combined with paracetamol	25,279 (1.4)
**10**	R01AD09, Mometasone	21,771 (1.2)	A02BC01, Omeprazole	28,943 (1.6)	B03BA01, Cyanocobalamine	26,776 (1.5)	A02BC01, Omeprazole	23,402 (1.3)
**11**	M01AB05, Diklofenak	21,517 (1.2)	A03FA01, Metoclopramide	27,871 (1.6)	N06AB06, Sertraline	25,347 (1.4)	C07AG01, Labetalol	22,749 (1.3)
**12**	G03AC09, Desogestrel	20,948 (1.2)	J01XE01, Nitrofurantoin	26,093 (1.5)	R05FA02, Opium derivatives and expectorants***	24,870 (1.4)	N06AB06, Sertraline	20,490 (1.2)

**13**	A02BC01, Omeprazole	20,901 (1.2)	N06AB06, Sertraline	25,976 (1.5)	N02AJ06, Codeine combined with paracetamol	21,852 (1.2)	R05FA02, Opium derivatives and expectorants***	18,494 (1.1)
**14**	R05FA02, Opium derivatives and expectorants***	19,183 (1.1)	G03GA08, Choriogonadotropin alfa	25,140 (1.4)	B03BB01, Folic acid	21,154 (1.2)	R06AD52, Promethazine combined with ephedrine and caffeine	18,224 (1.0)
**15**	J02AC01, Fluconazole	18,292 (1.0)	R05FA02, Opium derivatives and expectorants***	16,674 (0.9)	R05CB10, Bromhexine combined with ephedrine	20,288 (1.2)	B03BB01, Folic acid	16,561 (0.9)
**16**	R06AX27, Desloratadine	17,860 (1.0)	R01AD09, Mometasone	16,181 (0.9)	R01AD09, Mometasone	19,631 (1.1)	R05CB10, Bromhexine combined with ephedrine	15,325 (0.9)
**17**	M01AE02, Naproxen	17,191 (1.0)	B03BA01, Cyanocobalamine	15,995 (0.9)	R01BA01, Phenylpropanolamine	19,120 (1.1)	R01AD09, Mometasone	14,260 (0.8)
**18**	G03GB02, Clomifene	16,966 (1.0)	G03GA05, Follitropin alfa	15,449 (0.9)	R06AA04, Clemastine	18,078 (1.0)	R01BA01, Phenylpropanolamine	13,836 (0.8)
**19**	B03BB01, Folic acid	16,410 (0.9)	H01CC01, Ganirelix	14,665 (0.8)	R03BA02, Budesonide	16,414 (0.9)	B01AC06, Acetylsalicylic acid	13,193 (0.7)
**20**	N02AJ06, Codeine combined with paracetamol	15,767 (0.9)	R03BA02, Budesonide	13,912 (0.8)	R03AC03, Terbutaline	16,346 (0.9)	B01AB04, Dalteparin	12,730 (0.7)

Abbreviations: ATC, Anatomical Therapeutical Classification
*Displayed as number and proportion of the total number of pregnancies in the study population (n=1,761,183) with at least one dispensed prescription in each respective pregnancy period
**Pre-pregnancy refers to 90 days preceding start of pregnancy
*** A cough syrup containing ethylmorphine, cocillana and senega

Supplementary Table 6. Top 20 most commonly dispensed prescription medications during pregnancy in Sweden 2007–2022 by category of maternal age at delivery

	Category of maternal age at delivery, years (total number of pregnancies)													
	<20 (n=20,068)		*20–24 (n=201,883)*		*25–29 (n=536,111)*		*30–34 (n=625,376)*		*35–39 (n=324,010)*		*40–44 (n=71,002)*		*≥45 (n=4,726)*	
	**ATC, substance**	**No. (%)**	**ATC, substance**	**No. (%)**	**ATC, substance**	**No. (%)**	**ATC, substance**	**No. (%)**	**ATC, substance**	**No. (%)**	**ATC, substance**	**No. (%)**	**ATC, substance**	**No. (%)**
**1**	J01CA08, Pivmecillinam	2,463 (12.3)	R06AD52, Promethazine combined with ephedrine and caffeine	20,715 (10.3)	R06AD52, Promethazine combined with ephedrine and caffeine	57,583 (10.9)	R06AD52, Promethazine combined with ephedrine and caffeine	63,455 (10.3)	R06AD52, Promethazine combined with ephedrine and caffeine	31,236 (9.8)	H03AA01, Levothyroxine sodium	7,917 (11.4)	G03DA04, Progesterone	1,480 (33.4)
**2**	J01XE01, Nitrofurantoin	1,715 (8.6)	J01CA08, Pivmecillinam	19,065 (9.5)	J01CE02, Phenoximethyl-penicillin	37,437 (7.1)	J01CE02, Phenoximethyl-penicillin	50,328 (8.2)	J01CE02, Phenoximethyl-penicillin	28,440 (8.9)	G03DA04, Progesterone	7,891 (11.3)	G03CA03, Estradiol	1,246 (28.1)
**3**	R06AD52, Promethazine combined with ephedrine and caffeine	1,506 (7.5)	J01CE02, Phenoximethyl-penicillin	13,197 (6.6)	J01CA08, Pivmecillinam	36,173 (6.8)	H03AA01, Levothyroxine sodium	40,138 (6.5)	H03AA01, Levothyroxine sodium	27,986 (8.8)	R06AD52, Promethazine combined with ephedrine and caffeine	6,332 (9.1)	B01AC06, Acetylsalicylic acid	1,107 (25.0)
**4**	J01CE02, Phenoximethyl-penicillin	1,297 (6.5)	J01XE01, Nitrofurantoin	12,917 (6.4)	R06AD02, Promethazine	30,441 (5.7)	R06AD02, Promethazine	37,576 (6.1)	R06AD02, Promethazine	21,422 (6.7)	J01CE02, Phenoximethyl-penicillin	5,988 (8.6)	H03AA01, Levothyroxine sodium	747 (16.9)
**5**	R06AD02, Promethazine	997 (5.0)	R06AD02, Promethazine	11,209 (5.6)	H03AA01, Levothyroxine sodium	25,277 (4.8)	J01CA08, Pivmecillinam	35,237 (5.7)	J01CA08, Pivmecillinam	18,071 (5.7)	B01AC06, Acetylsalicylic acid	5,907 (8.5)	R06AD52, Promethazine combined with ephedrine and caffeine	447 (10.1)
**6**	A02BC01, Omeprazole	701 (3.5)	A02BC01, Omeprazole	7,007 (3.5)	J01XE01, Nitrofurantoin	24,685 (4.7)	J01XE01, Nitrofurantoin	24,781 (4.0)	G03DA04, Progesterone	17,502 (5.5)	R06AD02, Promethazine	5,163 (7.4)	R06AD02, Promethazine	421 (9.5)
**7**	R06AA04, Clemastine	635 (3.2)	N02BE01, Paracetamol	6,919 (3.5)	N02BE01, Paracetamol	18,866 (3.6)	N02BE01, Paracetamol	22,496 (3.6)	N02BE01, Paracetamol	14,147 (4.4)	J01CA08, Pivmecillinam	4,491 (6.5)	J01CE02, Phenoximethylpe nicillin	362 (8.2)
**8**	B03BB01, Folic acid	538 (2.7)	H03AA01, Levothyroxine sodium	6,717 (3.4)	A02BC01, Omeprazole	17,107 (3.2)	R05FA02, Opium derivatives and expectorants**	21,732 (3.5)	R05FA02, Opium derivatives and expectorants**	13,981 (4.4)	N02BE01, Paracetamol	3,993 (5.7)	J01CA08, Pivmecillinam	355 (8.0)
**9**	J01DB05, Cefadroxil	534 (2.7)	R06AA04, Clemastine	5,973 (3.0)	N02AJ06, Codeine combined with paracetamol	15,167 (2.9)	A02BC01, Omeprazole	19,870 (3.2)	J01XE01, Nitrofurantoin	13,602 (4.3)	J01XE01, Nitrofurantoin	3,454 (5.0)	B03BB01, Folic acid	350 (7.9)

**10**	N02BE01, Paracetamol	533 (2.7)	N02AJ06, Codeine combined with paracetamol	5,783 (2.9)	R06AA04, Clemastine	14,875 (2.8)	B01AC06, Acetylsalicylic acid	18,406 (3.0)	B01AC06, Acetylsalicylic acid	13,003 (4.1)	R05FA02, Opium derivatives and expectorants**	3,424 (4.9)	A02BC01, Omeprazole	346 (7.8)
**11**	B03BA01, Cyano-cobalamine	515 (2.6)	B03BA01, Cyano-cobalamine	5,169 (2.6)	B03BA01, Cyano-cobalamine	14,432 (2.7)	G03DA04, Progesterone	17,958 (2.9)	A02BC01, Omeprazole	12,171 (3.8)	A02BC01, Omeprazole	3,336 (4.8)	B01AB04, Dalteparin	342 (7.7)
**12**	J01CA04, Amoxicillin	436 (2.2)	B03BB01, Folic acid	5,049 (2.5)	R05FA02, Opium derivatives and expectorants**	14,122 (2.7)	B03BA01, Cyano-cobalamine	17,208 (2.8)	B03BA01, Cyano-cobalamine	10,876 (3.4)	B03BA01, Cyano-cobalamine	3,096 (4.4)	N02BE01, Paracetamol	331 (7.5)
**13**	H03AA01, Levothyroxine sodium	435 (2.2)	A03FA01, Metoclopramide	4,696 (2.3)	B03BB01, Folic acid	13,349 (2.5)	N02AJ06, Codeine combined with paracetamol	17,188 (2.8)	R01AD09, Mometasone	10,372 (3.3)	B03BB01, Folic acid	3,000 (4.3)	H02AB06, Prednisolone	329 (7.4)
**14**	N06AB06, Sertraline	428 (2.1)	R05FA02, Opium derivatives and expectorants**	3,996 (2.0)	R05CB10, Bromhexine combined with ephedrine	11,675 (2.2)	R06AA04, Clemastine	17,141 (2.8)	R05CB10, Bromhexine combined with ephedrine	10,248 (3.2)	G03GA08, Choriogonado-tropin alfa	2,932 (4.2)	C07AG01, Labetalol	326 (7.4)
**15**	R05CB10, Bromhexine combined with ephedrine	387 (1.9)	R05CB10, Bromhexine combined with ephedrine	3,859 (1.9)	B01AC06, Acetylsalicylic acid	11,658 (2.2)	R01AD09, Mometasone	16,828 (2.7)	B03BB01, Folic acid	10,032 (3.1)	R05CB10, Bromhexine combined with ephedrine	2,612 (3.8)	B03BA01, Cyano-cobalamine	252 (5.7)
**16**	A03FA01, Metoclopramide	383 (1.9)	J01DB05, Cefadroxil	3,684 (1.8)	A03FA01, Metoclopramide	11,252 (2.1)	R05CB10, Bromhexine combined with ephedrine	16,435 (2.7)	N02AJ06, Codeine combined with paracetamol	9,958 (3.1)	G03CA03, Estradiol	2,471 (3.5)	J01XE01, Nitrofurantoin	251 (5.7)
**17**	B03AA07, Ferrous sulfate	376 (1.9)	N06AB06, Sertraline	3,439 (1.7)	R01AD09, Mometasone	11,142 (2.1)	R01BA01, Phenyl-propanolamine	16,199 (2.6)	R01BA01, Phenyl-propanolamine	9,620 (3.0)	R01AD09, Mometasone	2,403 (3.5)	B01AB05, Enoxaparin	241 (5.4)
**18**	N02AJ06, Codeine combined with paracetamol	357 (1.8)	R03AC03, Terbutaline	3,373 (1.7)	N06AB06, Sertraline	10,155 (1.9)	B03BB01, Folic acid	16,139 (2.6)	R06AA04, Clemastine	9,541 (3.0)	N02AJ06, Codeine combined with paracetamol	2,294 (3.3)	R05FA02, Opium derivatives and expectorants**	225 (5.1)
**19**	R03AC03, Terbutaline	353 (1.8)	G01AA10, Clindamycin	3,219 (1.6)	R01BA01, Phenyl-propanolamine	9,679 (1.8)	N06AB06, Sertraline	13,093 (2.1)	G03GA08, Choriogonado-tropin alfa	8,562 (2.7)	R06AA04, Clemastine	2,209 (3.2)	R05CB10, Bromhexine combined with ephedrine	219 (4.9)
**20**	G01AA10, Clindamycin	343 (1.7)	R01AD09, Mometasone	3,107 (1.6)	R03AC03, Terbutaline	8,930 (1.7)	R03AC03, Terbutaline	11,022 (1.8)	N06AB06, Sertraline	7,546 (2.4)	C07AG01, Labetalol	2,189 (3.1)	R06AA04, Clemastine	162 (3.7)

Abbreviations: ATC, Anatomical Therapeutical Classification
*Displayed as number and proportion of total number of pregnancies in each category of maternal age with at least one dispensed prescription during pregnancy

Supplementary Figure 1 A-F. Proportion of pregnancies in Sweden 2007–2022 with at least one dispensed prescription within specific medication groups of interest during pregnancy (left) and in the three months postpartum (right) for antihypertensives (A and B), antidiabetics (C and D), antirheumatic medications (E and F). Medication groups defined in Supplementary Table 3.

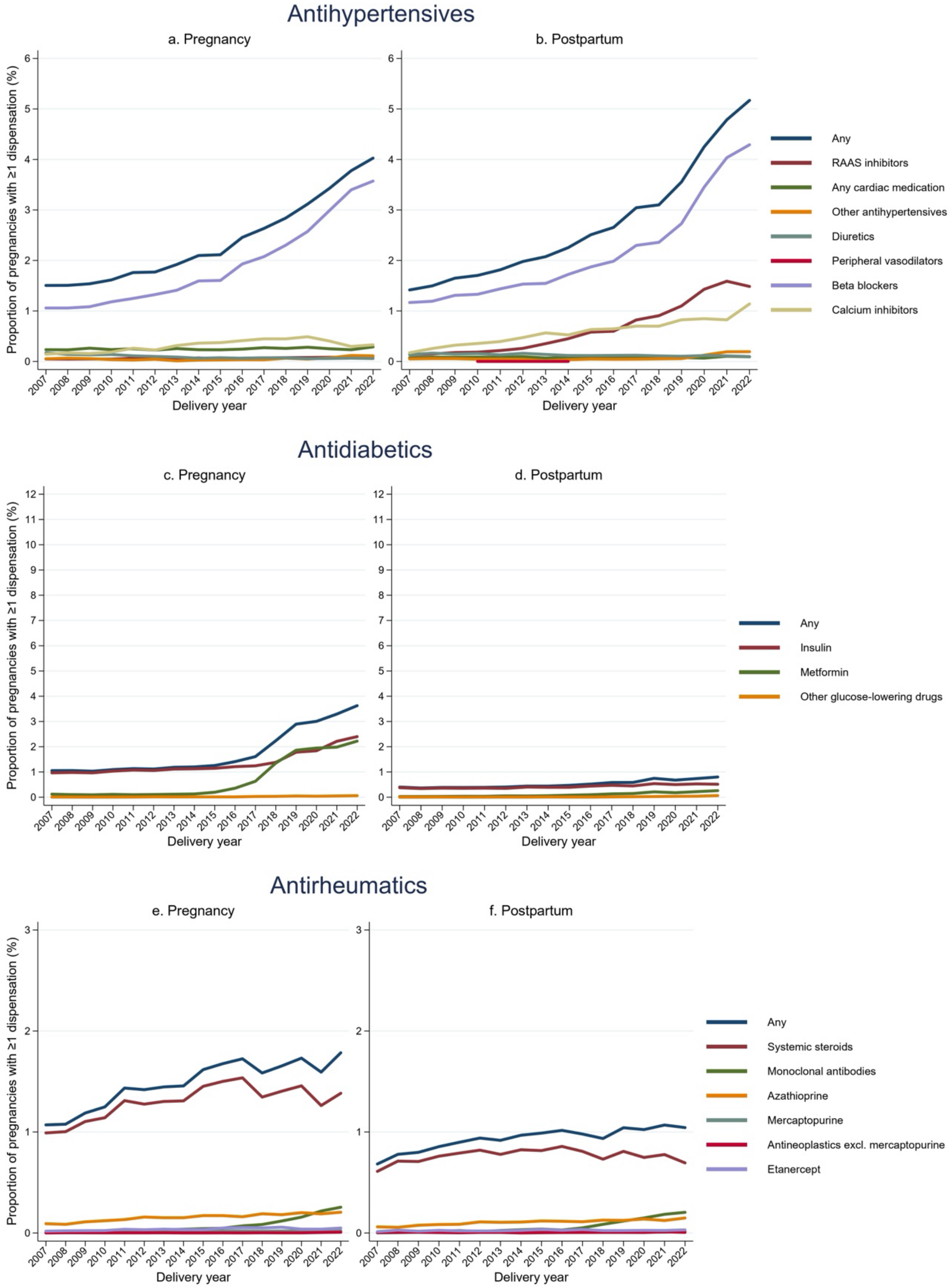

Abbreviations: RAAS, Renin-Angiotensin-Aldosterone System

Note: the data sources included do not capture over-the-counter medication or medication administered in hospital.

Supplementary Figure 2 A-F. Proportion of pregnancies in Sweden 2007–2022 with at least one dispensed prescription within specific medication groups of interest during pregnancy (left) and in the three months postpartum (right) for anti-infectives (A and B), asthma medications (C and D) and prescribed supplements (E and F). Medication groups defined in Supplementary Table 3.

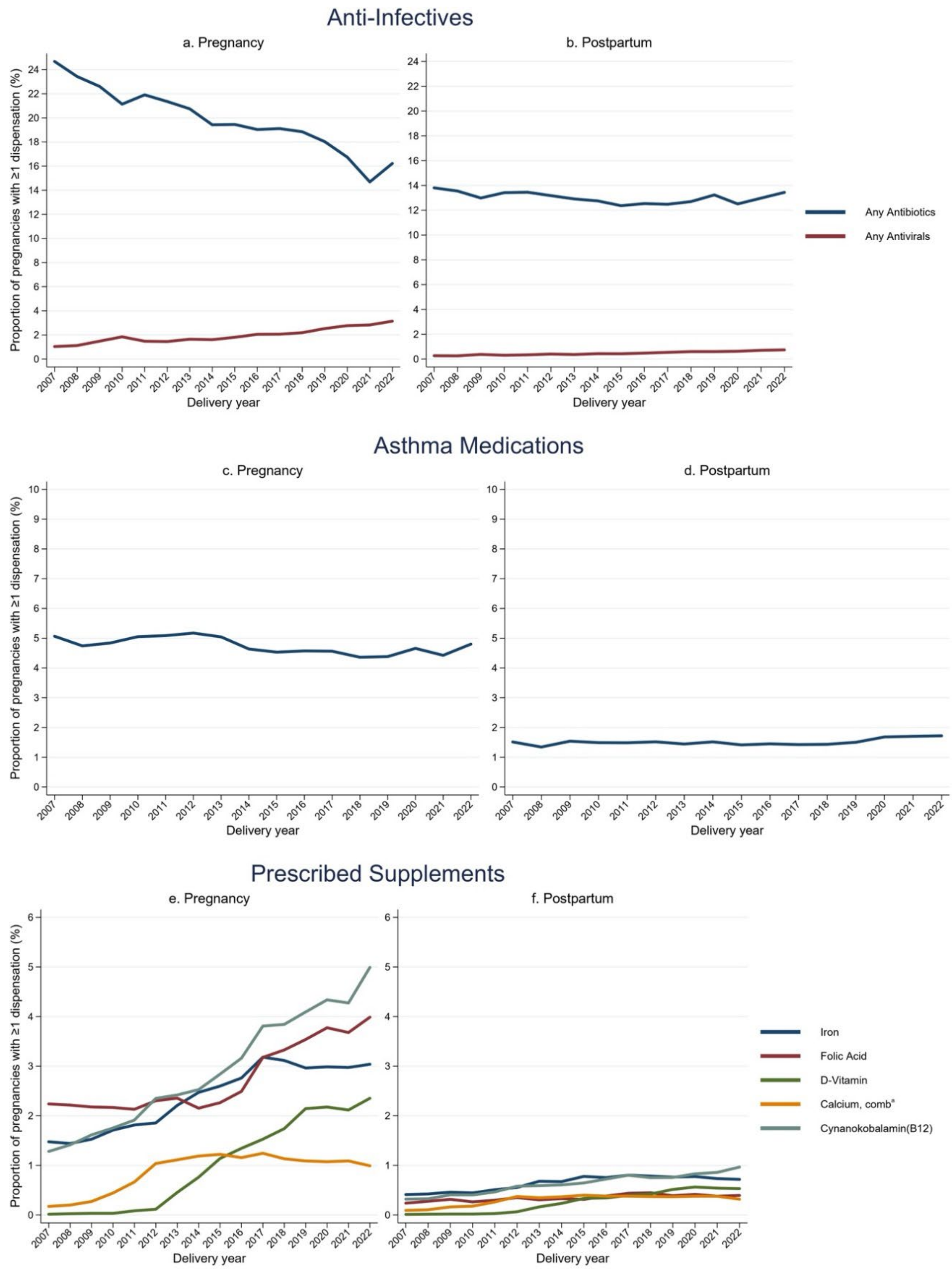

[a] Calcium, combinations with vitamin D and/or other drugs

Note: The data sources included do not capture over-the-counter medication or medication administered in hospital.

Supplementary description of results for specific medication groups of interest during pregnancy and in the three months postpartum

Amongst the ten medication groups with specific clinical interest, the most commonly dispensed during pregnancy were: antibiotics (348,744 women; 19.8%), antiemetics (263,755; 15.0%), and analgesics (145,664; 8.3%), and during postpartum were: antibiotics (229,113; 13.0%), analgesics (170,298; 9.7%), and psychotropic medications (74,160; 4.2%) (**Figure 5, Supplementary Figures 1-2).**

Psychotropics

Overall, 112,016 (6.4%) women dispensed a psychotropic medicine during pregnancy, and 74,160 (4.2%) postpartum, with an increasing trend over time. Antidepressants were the most commonly dispensed psychotropic medications during pregnancy (79,828, 4.5%) and postpartum (58,664, 3.3%), and use during pregnancy increased from 2.5% to 6.7% over the study period. The second most common psychotropics were anxiolytics, hypnotics and sedatives, with a stable proportion of pregnant women dispensing them over time during pregnancy (2%) and postpartum (1%). Less than 1% of pregnant women dispensed antipsychotics, lithium, or ADHD medication during pregnancy or postpartum **(Figure 5A-B)**.

Anticoagulants

During the study period, 77,565 (4.4%) of the women filled a prescription of any anticoagulant during pregnancy and 72,102 (4.1%) postpartum. However, there was an exponential increase of filled prescriptions of acetylsalicylic acid (ASA) during pregnancy, from 0.8% of pregnant women in 2007 to 10.3% in 2022 filling a prescription of ASA **(Figure 5C-D).**

Analgesics

Overall, 145,664 (8.3%) women dispensed an analgesic during pregnancy and 170,298 (9.7%) postpartum. The proportion of women with a dispensed prescription during pregnancy was stable across the study period, but postpartum there was a decrease from 11.3% in 2013 to 6.2% 2022, mainly driven by a decreased proportion of women dispensing paracetamol and non-steroidal anti-inflammatories (NSAIDs). The proportion of women who filled prescriptions of opiates other than codeine combinations decreased from over 2% to below 1%, during both pregnancy and postpartum **(Figure 5E-F).**

Antiemetics

In total, 263,755 (15.0%) women filled a prescription of an antiemetic during pregnancy, with the most common antiemetics being promethazine alone or in combination with caffeine and ephedrine, the prescription fills of which increased steadily over the study period. The use of other antiemetics than promethazine was generally low, as was use of any antiemetics postpartum (n=12,362, 0.7%) **(Figure 5G-H).**

Antihypertensives

During the study period, 42,041 (2.4%) of the women filled a prescription of any antihypertensive medications during pregnancy and 47,948 (2.7%) postpartum. Prescription fills of antihypertensive medications during pregnancy and postpartum increased from 1.5% and 1.4% in 2007 to 4.0% and

5.2% in 2022, respectively, mainly consisting of an increased prescription fills of beta receptor inhibitors **(Supplementary Figure 1A-B).**

Antidiabetics

During the study period 31,087 (1.8%) women filled a prescription of an antidiabetic medication during pregnancy and 9,208 (0.5%) postpartum, with a notable increase in proportion of women with prescription fills of especially non-insulin antidiabetics during pregnancy in the latest years, from around 0.1% before 2015, to 2,322 of 102,999 (2.3%) in 2022. A similar trend was observed postpartum, but the proportions were lower **(Supplementary Figure 1C-D).**

Antirheumatics

During the study period, 26,163 (1.5%) of the women filled a prescription of an antirheumatic medication during pregnancy and 16,481 (0.9%) postpartum, the majority of which were systemic steroids. The proportion of women with a dispensed prescription of monoclonal antibodies increased since 2017 both during pregnancy and postpartum, but were still below 0.3% at the end of the study period **(Supplementary Figure 1E-F).**

Antibiotics and antivirals

During the study period, 348,744 (19.8%) of the women filled an antibiotic prescription during pregnancy and 229,118 (13.0%) postpartum, whereas only 34,267 (1.9%) filled a prescription of an antiviral during pregnancy and 8,254 (0.5%) postpartum. The number and proportion of filled antibiotic prescriptions during pregnancy decreased from 25,471 of 103,202 (24.7%) in 2007 to 16,708 of 102,999 (16.2%) in 2022, and the most commonly prescribed antibiotics were pivmecillinam and phenoxymethylpenicillin. **(Supplementary Figure 2A-B)**

Other medication groups

During the study period, 83,504 (4.7%) of the women filled a prescription of an asthma medication during pregnancy and 26,627 (1.5%) postpartum, with no significant change in prescription rate during the study period. The prescription rates of cyanocobalamin (vitamin B12), folic acid, D-vitamin, calcium combined with D-vitamin and iron during pregnancy increased during the study period, but were all generally low, less than 5% during pregnancy and less than 1% postpartum. **(Supplementary Figure 2C-F)**
